# Detection of Epithelial-Mesenchymal Transition Status of Circulating Tumor Cells in Patients with Esophageal Squamous Carcinoma

**DOI:** 10.1155/2018/7610154

**Published:** 2018-06-26

**Authors:** Dingpei Han, Kai Chen, Jiaming Che, Junbiao Hang, Hecheng Li

**Affiliations:** Department of Thoracic Surgery, Ruijin Hospital, Shanghai Jiaotong University School of Medicine, China

## Abstract

**Purpose:**

To investigate the correlation between the status of epithelial-mesenchymal transition (EMT) of circulating tumor cells (CTCs) and esophageal squamous cell carcinoma (ESCC).

**Methods:**

The demographic data and blood samples of 21 patients with ESCC were collected retrospectively. CTCs were enriched by using optimized CanPatrolTM CTC enrichment technique. CTCs were identified and characterized according to the EMT markers (e-CTCs: epithelial CTCs; mix-CTCs: epithelial-mesenchymal-mixed CTCs; m-CTCs: mesenchymal CTCs). The correlation between CTCs and demographic data was analyzed.

**Results:**

Total 129 CTCs were detected in all the patients: 11(8.5%) CTCs of them were e-CTCs, 76(58.9%) were mix-CTCs, and 42(32.6%) were m-CTCs. The average number of CTCs from each patient was 6.1 ± 7.1 which included 0.5 ± 0.9 of e-CTCs, 3.6 ± 5.2 of mix-CTCs, and 2.0 ± 2.7 of m-CTCs; the difference between the three groups was significant (*P* = 0.017): the number of total CTCs was correlated with the number of mix-CTCs (*R*2 = 0.883, *P* < 0.01) and m-CTCs (*R*2 = 0.639, *P* < 0.01) but not e-CTCs (*R*2 = 0.012, *P* = 0.641) and the number of CTCs was correlated with the N stage and TNM stage in this study (*R*2 = 0.698 and *R*2 = 0.359).

**Conclusions:**

Mix-CTCs and m-CTCs might play an important role in progression of ESCC; the number of CTCs in ESCC might have the potential to be a predictor of prognosis.

## 1. Introduction

 Esophageal cancer ranks 5th in morbidity and 4th in mortality of all diagnosed cancers in China. Patients suffer great pain and undergo low quality of life after complex but so far most effective esophagectomy. In Asian, esophageal squamous cell carcinoma (ESCC) accounts for over 90% of all cases of esophageal cancer [1]; the poor prognosis is largely due to the rapid progress of local recurrences and metastasis. However, there are no specific tumor markers for early and small recurrences. In recent studies, the circulating tumor cells (CTCs) have been used as a “window” to monitor tumor prognosis [2]. In a case report, the CTCs counts in blood sample from a patient with esophageal cancer were in accordance with imaging scans at several time points [3]. With the advantage of accessible sampling, CTCs can investigate tumor progression in real time and monitor the response to further treatment [4].

Recurrence and metastasis are associated with multiple steps such as invasion, migration, and proliferation [5]. The epithelial-mesenchymal transition (EMT) in cancer metastasis is known to be regulated by several proteins that play important roles in invasion and migration of tumor cells [6]. During the process of EMT, the epithelial tumor cells lose their polarity which result in invasion into the surrounding tissues and penetration through vessel walls [7]. It is possible to detect the EMT status of CTCs in blood sample, just as what had been reported that aberrant activation of EMT was involved in the dissemination of tumor cells [8].

As we all know, during the EMT process of tumor cells, the expression of epithelial cell adhesion molecule (EpCAM) and cytokeratins (CK) will be downregulated, while the expression of twist and vimentin will be upregulated [9]. The EpCAM and CK are epithelial genes, and the vimentin and twist are mesenchymal genes. The mentioned genes are the common biomarkers of EMT and currently used as biomarkers of CTCs [10]. However, the EpCAM-based enrichment technique which is one of the most commonly used methods to isolating CTCs has limitation for detection of the subgroup of CTCs with accomplished EMT [11]. In recent years, a quantifiable dual-colorimetric RNA in situ hybridization assay was proposed and adopted in the research focused on EMT of CTCs in breast cancer [12]; there was no report on isolation of CTCs from patients with ESCC by this method. In this study, we firstly applied this enrichment technique (optimized CanPatrolTM CTC enrichment [13]) to isolate CTCs from blood samples of patients with ESCC, with the purpose of demonstrating the relationship between the EMT status of CTCs and ESCC.

## 2. Materials and Methods

### 2.1. Patients and Blood Samples

21 patients were recruited from January 2014 to June 2014 in Ruijin Hospital, Shanghai Jiaotong University School of Medicine. All the patients were diagnosed with ESCC by preoperative endoscopic biopsy and underwent esophagectomy with 2-field lymphadenectomy. The demographic data were shown in Table 1. We excluded the cases with the following criteria: patients with unresectable tumors, patients with preoperative chemotherapy or radiotherapy, and patients with ASA IV and V state. Blood samples of the 21 patients were collected at 1 day before surgery. We used the sample collection kit (Surexam, Guangzhou, China); the process of blood samples was as follows: 5 ml blood was collected in tubes with EDTA; after being shook up, the blood samples were transferred into the preservative tubes with ammonium chloride-based lysing buffer and then incubated for 30 minutes at room temperature.

### 2.2. CTC Enrichment

After being centrifuged, the blood samples in tubes were stratified. The top supernatant layer was discarded, and 5 ml PBS (Sigma, St. Louis, USA) was added to the remain cell suspensions. A filtration tube (Surexam, Guangzhou, China) with 8-micrometer-pore membrane filter (Millipore, Billerica, USA) was used. When the cell suspensions flew through the filter, the much larger CTCs were retained on the filter, while the other cells passed through. At last, the CTCs were fixed by 2% formaldehyde on the filters. A vacuum plate with valve settings (Surexam, Guangzhou, China) was applied in the filtration.

### 2.3. RNA In Situ Hybridization (ISH) Assay

The RNA-ISH method in this study was based on the branched DNA (bDNA) signal amplification technology [14]. The process of the RNA-ISH in this study was briefly as follows [13]: a 24-well plate (Corning, NY, USA) was used to treat the cells treated with a protease, then the hybridization was performed at 42°C for 2 hours, and the probes specific for the epithelial (EpCAM and CK8/18/19), mesenchymal (Vimentin and twist) and leukocyte (CD45) biomarker are shown in Table 1. After the unbound probes were washed out, the signal amplifications were performed at 42°C for 20 minutes with a cocktail preamplifier solution. Finally, the membranes were incubated with amplifier solution at 42°C for 20 minutes; then, the cells were stained by DAPI (Sigma, St. Louis, USA) for 5 minutes. The assay was analyzed by a fluorescence microscope (100x oil objective, Olympus BX53, Tokyo, Japan). The red dots of signal represented the epithelial biomarker expression, the green ones represented the mesenchymal biomarker expression, and the blue ones represented the leukocytes.

### 2.4. Statistical Analyses

The SPSS software program (Statistical Package for Social Sciences, vision 11.0) was used for statistical analysis. The measurement data were presented as mean ± SD and analyzed by the one-way ANOVA. The correlation analysis was analyzed by Pearson correlation coefficient. *P* < 0.05 is considered to be statistically significant.

## 3. Results and Discussion

### 3.1. Results

The demographic data of all the 21 patients are shown in Table 2. The mean size of tumors in this study was 16.9 ± 26.0 cm^3^; the mean length of specimens was 8.0 ± 1.9 cm.

**Figure 1:**
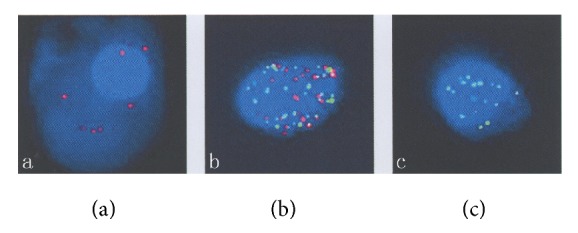
Three types of CTCs from ESCC (fluorescent microscope). (a) Epithelial CTC stained by epithelial markers (red dots); (b) epithelial-mesenchymal-mixed CTC stained by epithelial markers (red dots) and mesenchymal markers (green dots); (c) mesenchymal CTC stained by mesenchymal markers (green dots).

**Figure 2:**
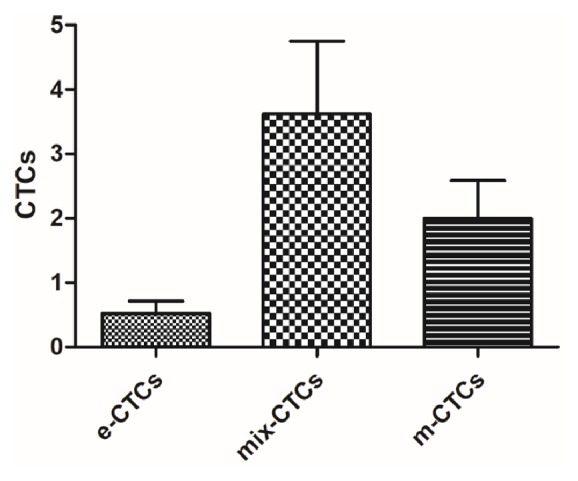
Histogram for average numbers of three types of CTCs from ESCC in this study (e-CTCs: epithelial CTCs; mix-CTCs: epithelial-mesenchymal-mixed CTCs; m-CTCs: mesenchymal CTCs).

**Figure 3:**
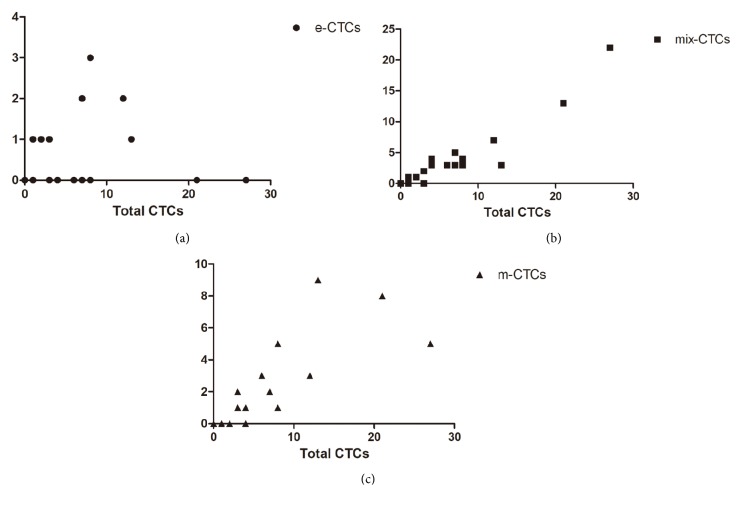
Tendency chart for Pearson correlation coefficient analysis between total CTCs and the three types of CTCs (e-CTCs: epithelial CTCs; mix-CTCs: epithelial-mesenchymal-mixed CTCs; m-CTCs: mesenchymal CTCs). (a) e-CTCs; (b) mix-CTCs; (c) m-CTCs.

As shown in Figure 1, three types of CTCs were detected in this study: epithelial CTCs (e-CTCs), epithelial-mesenchymal-mixed CTCs (mix-CTCs), and mesenchymal CTCs (m-CTCs). Total 129 CTCs were detected in all the patients, 11(8.5%) CTCs of them were e-CTCs, 76(58.9%) were mix-CTCs, and 42(32.6%) were m-CTCs. The average number of CTCs from each patient was 6.1 ± 7.1 which included 0.5 ± 0.9 of e-CTCs, 3.6 ± 5.2 of mix-CTCs, and 2.0±2.7 of m-CTCs; the difference between the three groups was significant (*P* = 0.017, Figure 2). In addition, as shown in Figure 3, the number of CTCs was correlated with the number of mix-CTCs (*R*^2^ = 0.883, *P* < 0.01, Figure 3(b)) and m-CTCs (*R*^2^ = 0.639, *P* < 0.01, Figure 3(c)) but not e-CTCs (*R*^2^ = 0.012, *P* = 0.641, Figure 3(a)).

As shown in Table 3, the results of correlation analysis showed that the number of CTCs was correlated with the N stage and TNM stage in this study (*R*^2^ = 0.698 and *R*^2^ = 0.359, respectively).

## 4. Discussion

Valuable biomarkers for detection of the tumor progression and treatment outcome are important in clinical cancer research [15], especially in ESCC which is accompanied by poor prognosis due to high incidences of metastasis. In clinical practice, we used computed tomography and serum tumor markers to investigate the progression of tumor in most cases, but these methods frequently lack sensitivity and specificity. In recent years, the function of CTCs in the tumor progression has been a research hotspot although the concept of CTCs was first recognized over 50 years ago [16]. With the development of molecular biological technique, many approaches have been developed to enrich CTCs in blood samples. The most widely used approach is CellSearch™ System [17], which has been applied in many types of cancer like breast, colorectal, and prostate cancer [18, 19]. However, the CellSearch™ System can only detect e-CTCs; it was reported that e-CTCs comprised just a small part (4.5%) of all CTCs in patients with hepatocellular [20]. The similar result was demonstrated in this study; only 8.5% of all CTCs was e-CTCs in ESCC. As the EMT is involved in the dissemination of tumor cells [8], we think that the conclusions from CellSearch™ System might have some limitations in the field of tumor progression; the similar result was reported in other study [21].

According to this study, in all the 129 CTCs from 21 patients with ESCC, 76(58.9%) were mix-CTCs and 42(32.6%) were m-CTCs, and the correlation analysis suggested that the number of CTCs was correlated with the number of mix-CTCs and m-CTCs. The results indicated that most CTCs from esophageal cancer had underwent EMT. It also suggested that EMT might play an important role in metastasis of ESCC, although the conclusion needs to be verified by study with more samples.

The ability to detect the CTCs at any stage of tumor provides a good method to diagnosis, monitoring, and prognostic evaluation [22, 23]. In this study, we detected the presence of CTCs in patients with T1 or N0 tumor, suggesting that EMT could occur in early stage of ESCC. This might be a reason for the high incidences of metastasis of ESCC, and it corresponded the fact that lymphatic metastasis could happen in T1 cases due to the abundant lymphatic network at submucosa.

The small sample size was a limitation of this study, and it might also cause bias in the statistical result. However, we found mix-CTCs of m-CTCs might be a predictor of prognosis for ESCC in this study; the result was evidenced by other publications that CTCs could predict the survival in ESCC [24], esophageal adenocarcinoma [25], and other malignancy such as prostate cancer [26]. Whether the different types of CTCs enriched by the method of this study would be a better predictor of prognosis for ESCC should be investigated by studies with more samples.

## 5. Conclusions

We found that the number of CTCs was correlated with ESCC progression although the sample size in this study was small. Mix-CTCs and m-CTCs might play an important role in EMT of ESCC, and the number of these CTCs in ESCC might have the potential to be a predictor of prognosis. The mechanism of promotion of EMT in CTCs from ESCC should be elucidated in further studies.

## Figures and Tables

**Table 1:** Capture probe sequences for the biomarkers.

Biomarker	Sequences (5'-3')
CD45	TCGCAATTCTTATGCGACTC
	TGTCATGGAGACAGTCATGT GTATTTCCAGCTTCAACTTC CCATCAATATAGCTGGCATT TTGTGCAGCAATGTATTTCC TACTTGAACCATCAGGCATC
EpCAM	TGGTGCTCGTTGATGAGTCA
	AGCCAGCTTTGAGCAAATGA AAAGCCCATCATTGTTCTGG CTCTCATCGCAGTCAGGATC TCCTTGTCTGTTCTTCTGAC CTCAGAGCAGGTTATTTCAG
CK8	CGTACCTTGTCTATGAAGGA
	ACTTGGTCTCCAGCATCTTG CCTAAGGTTGTTGATGTAGC CTGAGGAAGTTGATCTCGTC CAGATGTGTCCGAGATCTGG TGACCTCAGCAATGATGCTG
CK18	AGAAAGGACAGGACTCAGGC
	GAGTGGTGAAGCTCATGCTG TCAGGTCCTCGATGATCTTG CAATCTGCAGAACGATGCGG AAGTCATCAGCAGCAAGACG CTGCAGTCGTGTGATATTGG
CK19	CTGTAGGAAGTCATGGCGAG
	AAGTCATCTGCAGCCAGACG CTGTTCCGTCTCAAACTTGG TTCTTCTTCAGGTAGGCCAG CTCAGCGTACTGATTTCCTC GTGAACCAGGCTTCAGCATC
Vimentin	GAGCGAGAGTGGCAGAGGAC
	CTTTGTCGTTGGTTAGCTGG CATATTGCTGACGTACGTCA GAGCGCCCCTAAGTTTTTAA AAGATTGCAGGGTGTTTTCG GGCCAATAGTGTCTTGGTAG
Twist	ACAATGACATCTAGGTCTCC
	CTGGTAGAGGAAGTCGATGT CAACTGTTCAGACTTCTATC CCTCTTGAGAATGCATGCAT TTTCAGTGGCTGATTGGCAC TTACCATGGGTCCTCAATAA

**Table 2:** Demographic data.

Variables	N (%)/mean±SD
Gender	
male	17(80.9)
female	4(19.1)
Age	59.5 ± 8.2
BMI	23.2 ± 3.5
Tumor Location	
Middle	16(76.2)
lower	5(23.8))
Pathology	
Squamous	21(100)
Others	0(0)
Differentiation	
I	2(9.5)
I-II	1(4.8)
II	9(42.8)
II-III	6(28.6)
III	3(14.3)
pT Stage^*∗*^	
1	3(14.3)
2	4(19.0)
3	14(66.7)
pN Stage^*∗*^	
0	6(28.6)
1	8(38.1)
2	4(19.0)
3	3(14.3)
pTNM Stage^*∗*^	
I	3(14.3)
II	3(14.3)
III	12(57.1)
IV	3(14.3)

^*∗*^8th AJCC TNM staging.

**Table 3:** Correlation between total CTCs and demographic data.

Variables	*R* ^2^	*P* value
Gender	0.040	0.385
Age	0,009	0.687
BMI	0.013	0.627
Tumor location	0.049	0.336
Tumor size	0.026	0.487
T stage^*∗*^	0.151	0.082
N stage^*∗*^	0.698	<0.01
TNM stage^*∗*^	0.359	0.004
Differentiation	0.029	0.459

^*∗*^8th AJCC TNM staging.

## Data Availability

The date used to support the findings of this study are limitedly available from the first author upon request.
